# EDTA suppresses bacterial perseverance to 2-phenoxyethanol

**DOI:** 10.1128/spectrum.03807-25

**Published:** 2026-04-30

**Authors:** Yoshiko Miyahara, Takehisa Yano

**Affiliations:** 1 R&D—Safety Science Research, Kao Corporation, Ichikai, Tochigi, Japan; University of Manitoba, Winnipeg, Canada

**Keywords:** bacterial perseverance, 2-phenoxyethanol, EDTA, single-cell analysis, cosmetic preservatives

## Abstract

Perseverance is a phenotype in which a small subpopulation of bacteria continues to divide for hours in the presence of antimicrobial concentrations that inhibit bulk population growth, without acquiring genetic resistance. Perseverance has previously been reported only in antibiotic-treated bacteria, but not in preservative-treated bacteria. However, perseverance could limit preservative efficacy, and we investigated this phenomenon in preservative-treated bacteria. Cosmetic products rely on preservatives to control microbial growth; however, preservative types and concentrations are limited for safety reasons, and boosters are often added to enhance efficacy. Some formulations also contain nutrients, such as plant extracts, that promote heterogeneous microbial behavior. Therefore, single-cell analyses are needed to understand these responses and assess preservation risk. In this study, we combined time-lapse microscopy and a microdevice to perform single-cell analysis of *Staphylococcus ureilyticus*, a potential cosmetic contaminant, when exposed to the preservative 2-phenoxyethanol (PE) and booster ethylenediaminetetraacetate (EDTA). Conventional time-kill assays confirmed that EDTA enhanced the bactericidal effect of PE. Single-cell analysis revealed a subpopulation of cells that underwent up to five divisions over 23.4 h of PE exposure during viable count decline. Pedigree analysis showed that dividing lineages did not share cell fates with their ancestors, supporting a non-mutational perseverance phenotype. Compared with PE alone, the addition of EDTA markedly reduced single-cell division, with most cells dividing once or not at all despite similar population-level kill curves. These results demonstrate that single-cell analysis can reveal microbial behaviors masked in population-based assays and that EDTA may suppress persevering subpopulations that threaten product preservation.

**IMPORTANCE:**

This study highlights the significance of investigating the behavior of individual microbes in cosmetic products that employ limited microbial control strategies but may contain ingredients capable of supporting microbial proliferation. Through single-cell analysis of dynamic microbial responses to the preservative phenoxyethanol (PE) and booster ethylenediaminetetraacetate (EDTA), we demonstrate bacterial perseverance in the presence of PE and its suppression by EDTA. By enhancing the bactericidal effects of PE and suppressing perseverance, EDTA reinforces its role as a preservative booster. These findings advance our understanding of preservative performance and provide insights that may improve both the safety and effectiveness of preservatives in cosmetic products.

## INTRODUCTION

Antimicrobial exposure can elicit heterogeneous phenotypic responses within genetically identical bacterial populations. Persistence refers to the ability of a subpopulation to survive prolonged bactericidal exposure without acquiring genetic resistance, and in some systems, this state involves dynamically balanced division and death rather than complete growth arrest (1–3). More recently, Brandis et al. described a distinct phenotype termed perseverance, in which a minority subpopulation maintains active replication for several hours under growth-inhibitory concentrations without stable genetic changes (4). Unlike tolerance, which reflects delayed killing at the population level, perseverance is characterized by sustained cell division in a small fraction of cells despite inhibitory pressure. Together, these findings demonstrate that ongoing replication can occur in subpopulations even when bulk measurements indicate growth inhibition or population decline, underscoring the importance of single-cell approaches for resolving antimicrobial responses. These phenomena have been primarily reported in antibiotic-treated bacteria grown in nutrient-rich media, where inhibitory pressure is paired with available resources. Cosmetic formulations can produce similar microenvironments because preservatives impose antimicrobial stress. while formulation ingredients can supply nutrients or modulate microbial physiology. We therefore hypothesized that perseverance-like behaviors may also arise in cosmetic products.

Cosmetic products, such as skin lotions, creams, body soaps, and shampoos, are easily contaminated by environmental microorganisms as a result of repeated use. The absence of preservatives in these products allows microorganisms to survive and grow, which may pose health risks to users (5–7). Therefore, preservatives are typically incorporated into cosmetic products to reduce these risks. However, substances approved as preservatives are restricted to positive lists established through safety assessments in each country (8). The concentration of each preservative is also limited owing to safety concerns (9); therefore, sufficient preservative efficacy must be achieved using a limited range of substances and concentrations.

To overcome such challenges, preservative boosters are highly useful (9). These boosters themselves are not classified as preservatives, but they can enhance preservative efficacy when used in combination, often allowing for a reduction in the concentration of preservatives within formulations (10). Various compounds are incorporated as preservative boosters. For example, polyols, which are also used as moisturizers, exert booster effects by reducing the water activity required for microbial growth and by increasing the concentrations of preservatives in the aqueous phase, where they exert antimicrobial activity in emulsions (11, 12). Ethylhexylglycerin (EHG), a polyol with a relatively long chain length, has been shown to damage cell membranes even at low concentrations, thereby enhancing the activity of common preservatives such as methylparaben, methylisothiazolinone, and phenoxyethanol (PE) (13, 14). Indeed, the premixed ingredient “Euxyl PE 9010,” which combines PE with low levels of EHG, is widely used in cosmetic products (13). Additionally, chelating agents, which are included in formulations for various purposes such as maintaining product clarity and stabilizing colorants, have also been reported to act as preservative boosters by disturbing the cell surface structures and enhancing the efficacy of various preservatives (13, 15–19). Among these diverse combinations of preservatives and boosters, we recently found that combining PE with the chelator ethylenediaminetetraacetate (EDTA) markedly enhances preservative efficacy while inhibiting the induction of resistance to PE (15). Therefore, this combination was considered highly effective for achieving sufficient preservative efficacy in products; however, the mechanisms underlying these phenomena remain unclear.

An additional challenge for preservation arises from product composition. Cosmetic formulations often contain ingredients such as plant extracts, polysaccharides, amino acids, and minerals that can serve as nutrients or otherwise affect microbial physiology (20). In addition, there is a risk that preservative concentrations may locally or transiently fall below the minimum inhibitory concentration (MIC). For example, in manufacturing environments, residual water from equipment cleaning or condensation in mixing tanks may locally dilute preservatives. During product use, accidental introduction of water or the contamination associated with repeated consumer contact, including skin-derived compounds, may dilute preservatives. For these reasons, even when the concentration of preservatives in a product exceeds the MIC, such dilution events may cause the concentrations to locally or transiently drop below the MIC. Therefore, it is important to understand microbial behavior within the growth-inhibitory range and to determine appropriate preservative concentrations accordingly. Under such conditions, we hypothesized that these environments could allow small subpopulations to exhibit behaviors, including perseverance, that are not detected by conventional population-level assays. Consequently, assessing preservative performance solely using bulk colony counts or time-kill curves may miss critical single-cell behaviors relevant to product safety.

Various experimental approaches to investigate the mechanisms of antimicrobial action and microbial resistance have been developed; however, most studies have been conducted in the context of antibiotics rather than preservatives. These approaches have focused on physicochemical interactions between antibiotics and their cellular targets, physiological and biological changes within cells, and mutations that contribute to resistance (21–23). Moreover, recent advances in single-cell analysis techniques have revealed the characteristics and variability of individual cells within microbial populations (24). Notably, methodological approaches that combine microdevices with time-lapse microscopy have enabled the trapping and long-term tracking of microbial cells, allowing direct observation of single-cell behavior under drug exposure using fluorescent dyes and proteins (25–28). These methods have revealed specific phenomena in small subpopulations, such as persistence and perseverance, that are masked by population-level methods (1, 2, 4) and are therefore well suited to study heterogeneous responses in complex formulations.

To investigate these hypotheses, a combined approach using microdevices and time-lapse microscopy was considered particularly valuable. In this study, we focused on *Staphylococcus ureilyticus*, a microorganism that resides on human and animal skin and may contaminate cosmetic products (29, 30). We investigated the behavior of individual cells exposed to PE and EDTA in the presence of nutritional components to assess how EDTA affects preservative efficacy at the single-cell level.

## RESULTS

### Minimum inhibitory concentration (MIC) and minimum bactericidal concentration (MBC)

To assess the susceptibility of *S. ureilyticus* KMC153 to PE and EDTA, we determined the MICs and MBCs using cells harvested from agar plates to simulate environmental contamination of products and thereby include a mixture of exponential- and stationary-phase cells, as described in ISO11930, the standard method for preservative efficacy testing. Because preservatives are typically used at higher concentrations than antibiotics, and small differences in concentration can significantly affect their antimicrobial activity, we employed a 1.25-fold dilution series (0.2–2.0% for PE and 0.04–5.0% for EDTA). The MIC and MBC of PE were 1.3% and 1.6%, respectively. For EDTA, the MIC was 0.14%, whereas the MBC was >5.0%, which was the maximum concentration in our assay. The resultant difference between the MIC and MBC for PE was 0.3%, whereas that for EDTA was >4.8%. All experiments were performed independently in triplicate, yielding consistent results.

### Killing kinetics at the population level

**Fig 1:**
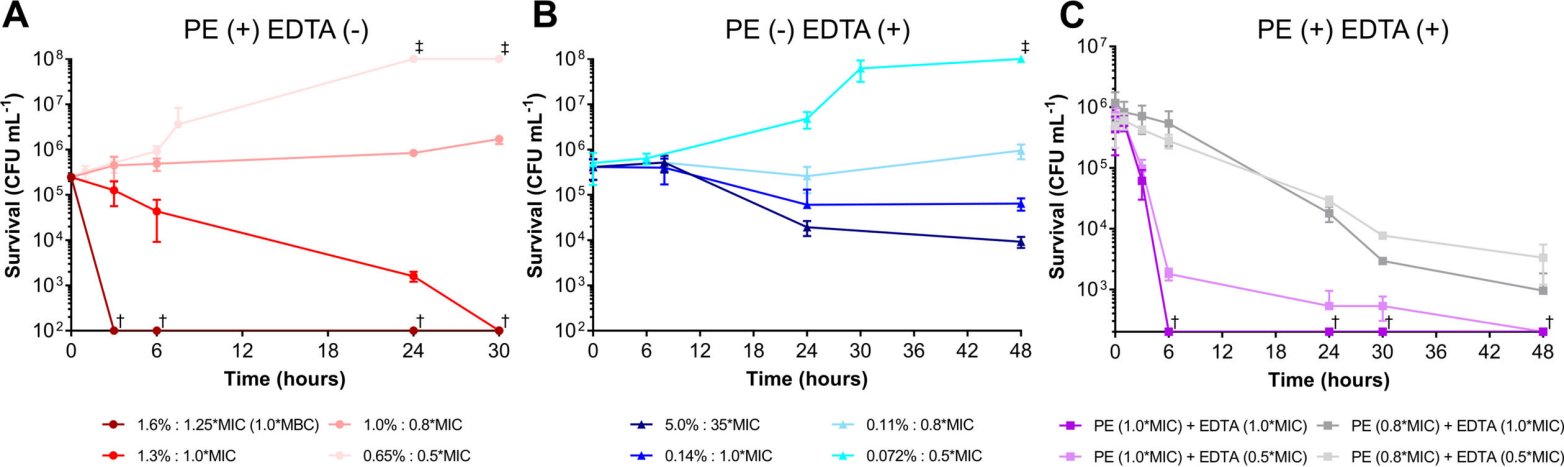
Time–kill curves of phenoxyethanol (PE) and ethylenediaminetetraacetic acid disodium (EDTA). Cultures were treated with PE (**A**), EDTA (**B**), or combinations of both (**C**) at the concentrations used for minimum inhibitory concentration measurements. Data points represent the mean ± standard deviation (SD) (*n* = 3). Dagger symbols indicate values below (†) or above (‡) the detection limits.

Some antibiotics with large differences between their MICs and MBCs exhibit different killing kinetics from those with similar values (31). To determine whether the bactericidal effects of PE and EDTA also differ, we incubated cells with these agents at concentrations near their MICs and MBCs and monitored viable counts over time. For PE, cell numbers increased at 0.8 × MIC (1.0%), whereas the number of viable cells gradually decreased to below the detection limit after 30 h at 1.0 × MIC (1.3%). At 1.25 × MIC (equivalent to 1.0 × MBC; 1.6), viable cell counts fell below the detection limit within 3.0 h (Fig. 1A), indicating bactericidal activity of PE under these conditions. For EDTA, the number of cells increased at 0.8 × MIC (0.11%). At 1.0 × MIC (0.14%), viable counts did not increase but were still detectable after 48 h. Furthermore, even at 35 × MIC (5.0%), the maximum concentration in the MBC assay, viable cells were still detected after 48 h (Fig. 1B), consistent with predominantly bacteriostatic activity of EDTA in our assay.

Next, we investigated the booster effects of EDTA. We considered that there are two types of such effects: (i) an accelerated reduction in viable cells when preservatives alone already decrease microbial population, and (ii) a shift from no reduction to a clear decrease when preservatives alone fail to reduce microbial numbers. To assess the first type, we combined 1.0 × MIC of PE, which reduced the number of viable cells below the detection limit after 30 h, with 1.0 × MIC of EDTA. This combination reduced viable cell counts to below the detection limit within 6.0 h. To evaluate the second type, we combined 0.8 × MIC of PE, which led to an increase in viable cell counts, with EDTA. This combination not only halted the increase in viable cells but also achieved a reduction of more than 2 log units (Fig. 1C).

### Killing kinetics at the single-cell level

**Fig 2:**
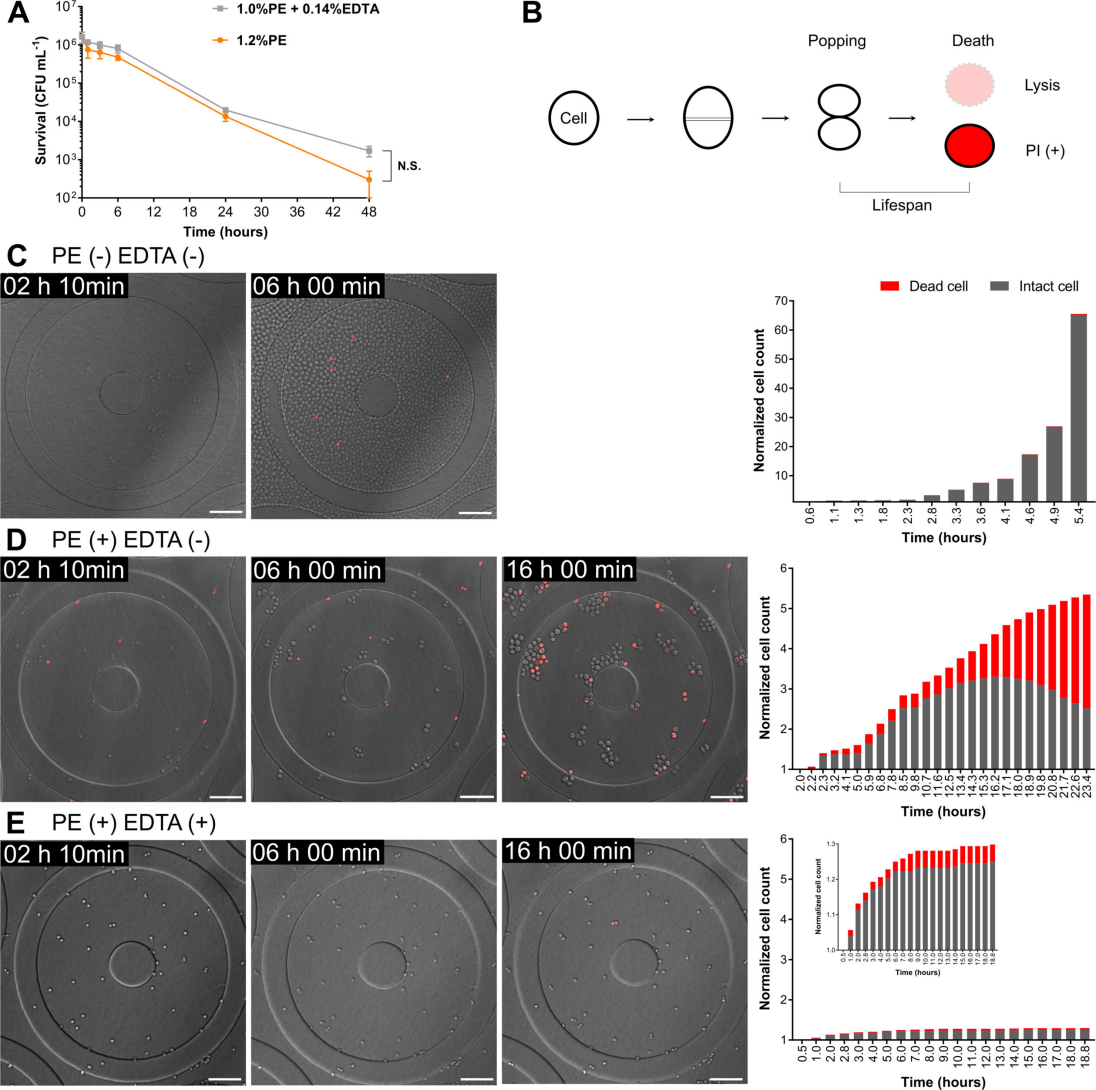
Single-cell analysis in a microdevice with cell trapping chambers. (**A**) Time–kill curves against 1.2% PE (orange) or 1.0% PE + 0.14% EDTA (gray), indicating the conditions used for single-cell analyses. Data points represent the mean ± SD (*n* = 3). N.S. indicates no statistical significance as determined using two-way repeated-measures analysis of variance (*P* = 0.4387). (**B**) Schematic diagram of the cell cycle. Cell emergence was detected as a popping event, while cell death was identified using propidium iodide (PI) staining or cell lysis. (**C–E**) Upper images show snapshots of time-lapse imaging. Dead cells were stained with PI, and the cells were imaged in both fluorescence and bright-field channels. Scale bar, 2 µm. Numbers at the upper left of images indicate elapsed time after the flow started (hh:mm). Lower graphs show normalized counts of intact and dead cells. For each assay, 32 (**C**), 239 (**D**), and 228 (**E**) cells were used as mother cells and were exposed to Soybean-Casein Digest (SCD) broth (**C**), 1.2% PE (**D**), or a combination of 1.0% PE and 0.14% EDTA (**E**), respectively. In panel **E**, a graph with the zoomed *y*-scale is included to more clearly display changes in cell counts.

To explore the combined effects of these compounds, we focused on the booster effect below the MIC because the transition from increasing to decreasing viable counts is critical for product preservation. Using time-kill experiments, we identified a concentration of PE alone (1.2%) that exhibited killing kinetics nearly identical to those observed with the PE + EDTA combination (1.0% and 1.4%, respectively) (Fig. 2A). Although 1.2% PE is slightly below the MIC (1.3%) determined in our MIC assay, it nevertheless prevented cell proliferation and induced a killing trend, indicating that this concentration lies within the growth-inhibitory range. Therefore, we performed subsequent single-cell analyses at this concentration.

In single-cell analyses, viable cells are generally detected using intracellular fluorescent proteins or dyes that reflect metabolic activity or membrane integrity (32). However, the fluorescence of such proteins or dyes may be influenced by aggregation or by interactions with preservatives or boosters, whose bactericidal effects may even interfere with fluorescence signals. Therefore, we used propidium iodide (PI) to selectively stain dead cells and identified live cells as those visible in bright-field microscopy but lacking PI fluorescence. Daughter cell emergence was detected by popping events (33), and cell death was detected by PI staining and lysis (Fig. 2B). The microdevice used here followed the design previously described (5, 28) with no major modifications. We first confirmed that the strain grew within the fabricated microdevice (Fig. 2C, upper panel). The cell number increased 65-fold over 5.4 h (doubling time: 36.3 min), with live cells accounting for 99.5% and dead cells for only 0.5% of the total population. After 6.0 h of culture, the cells overflowed from the chambers, making it impossible to track cell division and death at the single-cell level (Fig. 2C, lower panel). Therefore, subsequent analyses were conducted before the cells overflowed from the chambers.

Next, single-cell analysis was performed using PE alone or PE in combination with EDTA at the same concentrations as those shown in Fig. 2A. In the PE-alone condition (Fig. 2D and Movie S1), the number of live cells increased until 16.2 h and then decreased, whereas the total viable cells decreased throughout the exposure period on the time–kill curve. PI-positive or lysed cells began to appear 2 h after the start of the experiment, gradually increased in number, and accounted for more than half of the total cell population by 23.4 h. In contrast, under the combined treatment of PE and EDTA (Fig. 2E and Movie S2), the number of live cells increased slowly and slightly until 6.0 h, after which the growth plateaued. Notably, the increase in the total cell number by 18 h was only 27.3% of that observed under the PE-alone condition. PI-positive or lysed cells appeared as early as 1.0 h after the start of the experiment and gradually increased in number; however, even at 18.8 h, dead cells accounted for only 3.7% of the total population.

To assess the significance of this growth suppression, we investigated the concentration of PE required to achieve a comparable inhibitory effect on cell division in the absence of EDTA. The results showed that 1.25 × MIC (corresponding to the MBC, 1.6%) was necessary to reproduce this effect (Fig. S1).

### Cell division frequency at the single-cell level

**Fig 3:**
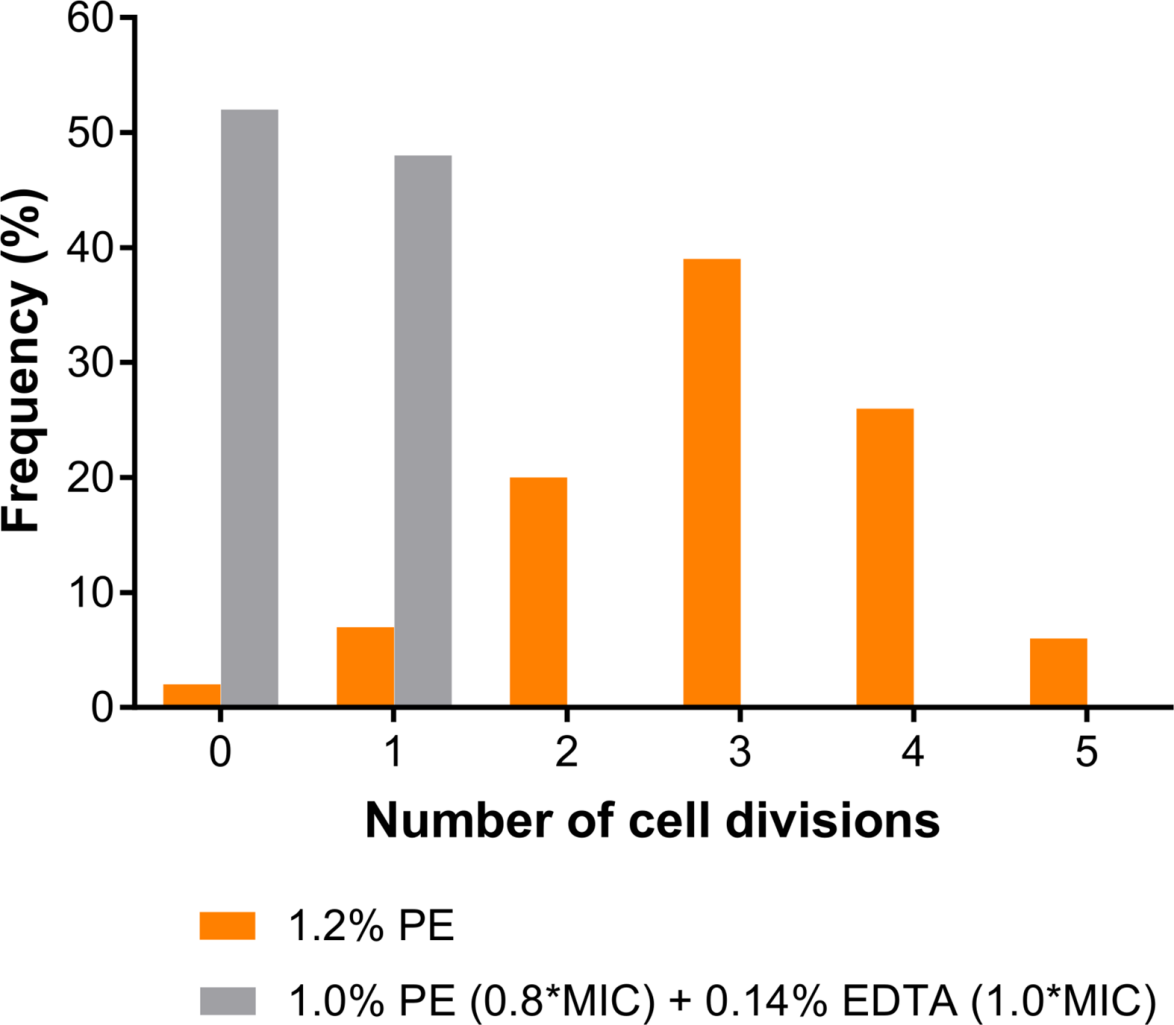
Distribution of the number of cell divisions. The number of divisions of the mother cells during treatment with PE alone (orange) or with the combination of PE and EDTA (gray) was quantified, and the frequency distribution is shown. The numbers of mother cells analyzed were 102 for 1.2% PE and 312 for 1.0% PE + 0.14% EDTA, respectively. A significant difference between PE treatment with and without EDTA, as determined using the Kolmogorov–Smirnov test (*P* < 0.0001).

To quantitatively evaluate growth suppression, we tracked each mother cell at the start of the experiment to determine the number of times it divided by the end of the observation period. Under the PE-alone treatment, some mother cells underwent up to five divisions over 23.4 h, with approximately 70% of all mother cells dividing three or more times (3.0 ± 1.1 cell divisions) and 2.0% of them dividing five times. In contrast, under the combined PE and EDTA treatment, no mother cells divided more than twice over 18.8 h, and most cells either did not divide at all or divided only once (0.5 ± 0.5 cell divisions; Fig. 3). Additionally, in the absence of PE and EDTA (Fig. 2C), the average number of divisions was 6 over 5.4 h. Notably, the proportion of cells that underwent division declined with each successive generation under PE treatment (Fig. S2).

### Characterization of the perseverance phenotype

**Fig 4:**
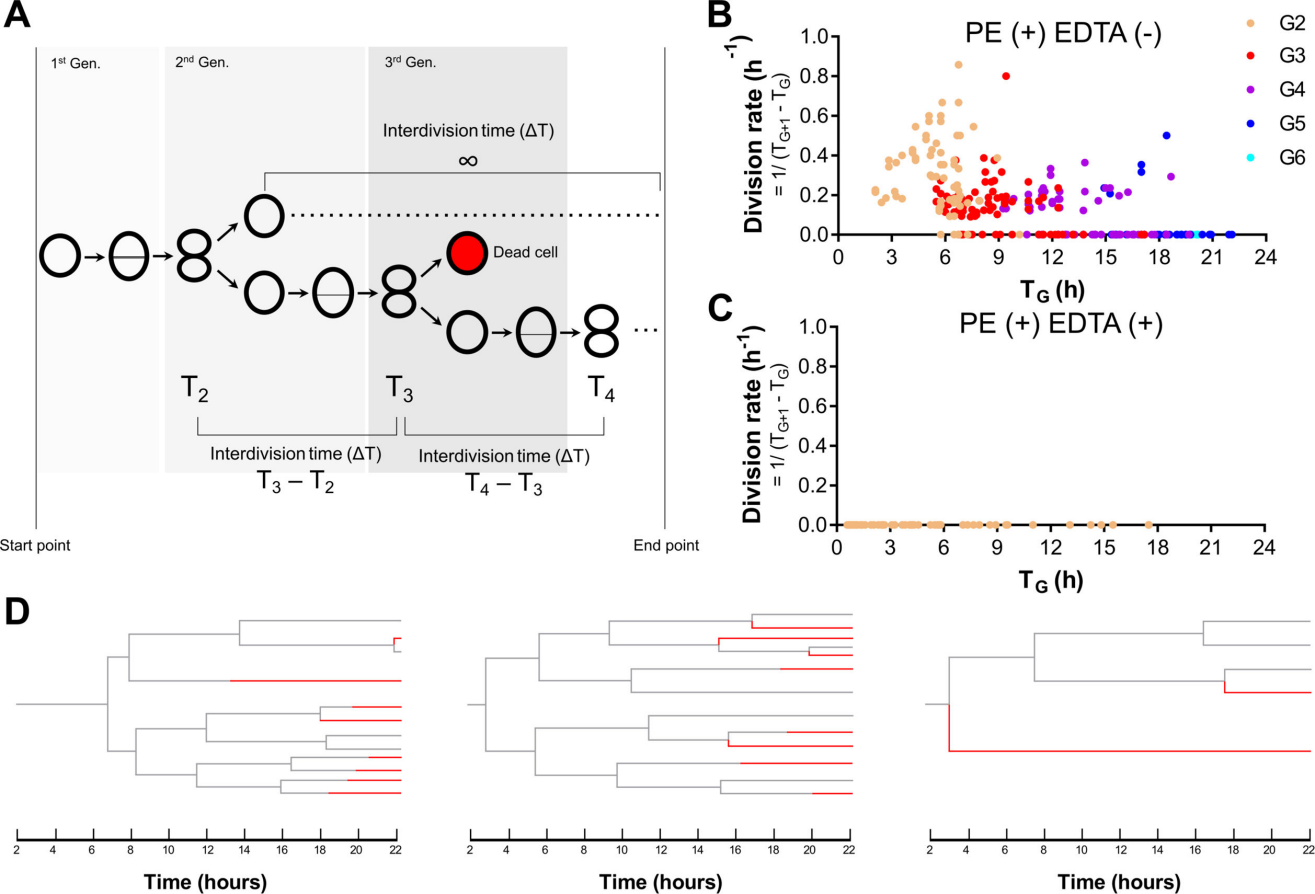
Perseverance phenotype and its suppression by EDTA. (**A**) Schematic diagram showing the method for measuring interdivision times for each generation. Given that the emergence times of the mother cells at the start of the experiments were unknown, interdivision times were measured beginning with the second generation. (**B and C**) Single-cell division rates, calculated as the reciprocal of interdivision times, were plotted against each cell’s time of emergence for cells exposed to 1.2% PE (**B**) and for cells exposed to the combination of 1.0% PE and 0.14% EDTA (**C**). The numbers of cells analyzed were 255 for 1.2% PE and 150 for 1.0% PE + 0.14% EDTA, respectively. Each color represents a distinct generation. (**D**) Pedigree analyses of cells exposed to 1.2% PE. The fates of three representative mother cells present at the initial time point (*T* = 2 h) were analyzed, and the corresponding pedigree trees are shown. Boundaries changing from gray to red indicate cell death.

Some mother cells continued to divide multiple times in the presence of 1.2% PE, despite decreases in viable cell counts, suggesting bacterial perseverance (4). To evaluate whether PE induced this phenotype, we analyzed temporal changes in single-cell division rates and the fates of progeny across generations. As staphylococci exhibit minimal elongation during division, division timing rather than elongation rate was used to quantify growth rate (Fig. 4A). Interdivision time (Δ*T*) was measured for each generation, and the division rate was defined as the reciprocal of Δ*T*. Cells that did not divide during the observation period were assigned a division rate of zero. Mother cells in the first generation (G1) were excluded from this analysis because their emergence times were unknown.

When exposed to either PE alone or the PE + EDTA combination, the timing of each cell’s emergence was plotted on the *x*-axis, and the division rates were plotted on the *y*-axis (Fig. 4B and C). The average division rates for generations G2–G5 were 0.30 ± 0.19, 0.14 ± 0.13, 0.085 ± 0.11, and 0.05 ± 0.13, respectively. With PE alone treatment, some cells did not divide; however, a subset of the population divided at a relatively consistent rate, with dividing cells observed up to 18.7 h after the start of exposure. The division rates within this subpopulation were not uniform, with the fastest dividing cells exhibiting rates more than eight times higher than those of the slowest (Fig. 4B). Among these, the fastest dividing cells had a division rate of 0.86 (h^−1^). For comparison, under conditions without PE—specifically in Soybean-Casein Digest (SCD) medium—the average division rate was 1.38 (h^−1^) (Fig. S3), indicating that division with PE alone occurred at a lower rate than those in SCD medium. In contrast, under the PE + EDTA combination treatment, mother cells (G1) divided at various times, with some dividing within 1.0 h after exposure began and others dividing 17.5 h later; however, no divisions were observed in cells other than the mother cells (Fig. 4C).

Next, pedigree analysis was conducted to investigate whether cells that underwent multiple divisions under PE treatment alone had acquired genetic resistance. As the proportion of cells exhibiting the perseverance phenotype decreased with each successive generation (Fig. S2), a total of 32 mother cells were selected to represent lineages with varying numbers of generations exhibiting this phenotype (Fig. 4D). The analysis revealed that even among first- and second-generation progeny derived from the same mother cell, some continued to divide while others died, indicating divergent fates within genetically related lineages (Fig. 4D).

## DISCUSSION

In this study, we performed single-cell analysis using cell-trapping microchambers to investigate microbial behavior under preservative exposure. Given our primary aim of directly comparing time-resolved population-level killing kinetics with single-cell dynamics, we measured colony-forming units (CFUs) and compared these population-level kinetics with single-cell behavior. We examined the responses of cells to the widely used preservative PE and its booster, EDTA. To the best of our knowledge, this study is the first to demonstrate that a subpopulation of cells continued to divide in the presence of PE within the growth-inhibitory range and that the addition of EDTA eliminated this subpopulation. The following discussion focuses on these phenomena in the context of cellular susceptibility to PE and EDTA.

Regarding cellular susceptibility to PE and EDTA, PE exhibited killing kinetics at 1.2%, consistent with previous reports indicating that a concentration of 1.25% demonstrates bactericidal efficacy against *Staphylococcus aureus* (ATCC 25923 and ATCC 29213) (34). In contrast, EDTA showed inhibitory activity at 0.14%, and even at the highest concentration tested (5%), the reduction in viable bacterial count was less than 2 log units after 48 h. For *S. aureus* (ATCC 6538), the reported MIC is 0.42% and the MBC is 1.69% (35). For methicillin-resistant *S. aureus* clinical strains, the MIC is 0.063% and the MBC is 1% (36), whereas for clinical strains of *S. epidermidis*, the MIC is 0.015% and the MBC exceeds 0.73% (37). Although MIC and MBC values vary significantly depending on species and strain, previous reports consistently demonstrate a large difference between the MIC and MBC, indicating bacteriostatic effects (37). A similar trend observed for *S. ureilyticus* in this study suggests that EDTA may exert bacteriostatic effects across a diverse range of microorganisms.

EDTA has been reported to chelate essential metal ions necessary for growth (38–40), as well as to destabilize cell envelopes by binding metal ions that stabilize cell components such as lipopolysaccharides and peptidoglycan (37, 39, 41–43). Although the booster effect of EDTA has been seldom investigated, this destabilization of the cell envelope may enhance the penetration of PE into the cell membrane. In this study, both types of booster effects were observed: one that enhances effects already exhibited and another that enables bactericidal effects not observed under individual treatments. These booster effects may reflect related physiological or physicochemical processes, but their exact basis remains to be determined.

For subpopulation analysis using the microdevice, we first examined how growth behaviors differed under the experimental conditions. The typical doubling time for *Staphylococcus* species is approximately 20 min (44), indicating that the observed 36.3 min in the microdevice was slightly slower. The reason for this delay requires further investigation, but one possibility is the mechanical stress imposed by the semi-permeable membrane covering the chamber, which restricts Brownian motion and bacterial motility and may reduce oxygen concentrations compared to standard culture conditions. However, as very few dead cells were observed in the absence of PE and EDTA, this stress likely does not significantly affect bacterial viability.

Next, we discuss the behaviors observed under PE treatment alone or in combination with EDTA treatments. For PE treatment alone, it is notable that viable cell counts decreased at the population level, while some cells continued to divide for a certain period at the single-cell level. Importantly, the perseverance phenotype appeared to be dependent on PE concentration and was suppressed at higher PE concentrations. As mutagenesis-driven resistant cells would be expected to divide throughout the incubation period, these dividing cells likely did not acquire resistance through mutation. Such resistant subpopulations without mutations have been reported to exhibit a perseverance phenotype (4). Although a detailed mutational analysis is necessary, the viable subpopulation in the PE treatment did not display a stable phenotype across generations in the pedigree analysis. To the best of our knowledge, this is the first report of the perseverance phenotype in the presence of preservatives. Limitations of this study include the analysis of only a single strain of *S. ureilyticus* and the observation of the perseverance phenotype only with PE, the sole preservative tested. Additional experiments using other preservatives and contaminating strains will be necessary to fully elucidate the conditions under which the perseverance phenotype arises in products.

Such surviving subpopulations could pose a significant threat to product preservation, and the suppression of perseverance by EDTA highlights its potential as a preservative booster. Exposure to an inhibitory concentration of PE produced mother cells that divided up to five times within 23.4 h, representing 2.0% of the population. This proportion is comparable to that reported previously; for example, in a study of perseverance with rifampicin, approximately 2% of the population was reported to divide up to nine to ten times (mean, 3.8 ± 1.2 divisions) under conditions at the MIC, with frequent mutagenesis occurring at loci unrelated to resistance (4). These mutations are assumed to arise spontaneously during replication, and their accumulation poses a risk of developing resistance, leading to decreased susceptibility to the antimicrobial agent. Although stable resistance associated with genetic mutations was not observed under the short experimental conditions employed in this study, prolonged exposure to preservatives could reduce susceptibility to PE. Indeed, *Staphylococcus* and *Pseudomonas* species have been reported to acquire resistance to PE through repeated exposure (15, 45). Furthermore, *Staphylococcus* species were reported to exhibit suppression of PE resistance development when treated with a combination of PE and EDTA (15). To determine whether perseverance contributes to PE resistance, or whether EDTA-mediated inhibition of perseverance can also suppress PE resistance, requires experiments under longer exposure conditions and comparative genomic analyses at the single-cell level on cells that have undergone multiple divisions. In contrast, elucidating the differences between cells exhibiting perseverance and those that do not, which exist prior to exposure to PE, is crucial for establishing a robust preservative system; however, these differences have not yet been clarified. The perseverance phenotype has been observed in response to antibiotics with different mechanisms of action, such as rifampicin and nitrofurantoin, as well as with PE, suggesting that characteristics of cell surface structures involved in drug permeability may play a role. Factors such as the chain length and degree of unsaturation of membrane fatty acids, as well as the polysaccharide composition of the peptidoglycan layer, may be relevant. These cell surface characteristics may change in response to growth phases and stress, potentially contributing to the perseverance phenotype independently of genetic factors. Further investigation of the mechanisms underlying perseverance in relation to PE requires isolating persevering and non-persevering cells and analyzing their differences through various approaches, including examination of cell membrane structures and polysaccharide composition.

EDTA suppressed cell division in the presence of PE, suggesting that it may be a prominent preservative booster that not only enhances the antibacterial activity of PE but also reduces the risk of microbial proliferation. Furthermore, single-cell analysis has proven effective for clarifying how preservatives and boosters affect individual cells. Our findings indicate that single-cell analysis can be used to validate optimal combinations and concentrations of various preservatives and boosters, guiding the design of preservative formulations that are both safe for humans and minimize the risk of microbial proliferation in cosmetic products. Future studies could extend these findings by analyzing multiple strains and preservatives, assessing the effects of long-term exposure, and exploring the molecular determinants of perseverance to inform the rational design of preservative formulations.

## MATERIALS AND METHODS

### Bacterial strain and growth conditions

*S. ureilyticus* KMC153 was isolated from the environment surrounding manufacturing equipment in Tokyo, Japan. The strain was identified through whole-genome sequencing, and the sequence was deposited in the DDBJ Sequence Read Archive (BioProject accession no. PRJDB38213). For all antimicrobial experiments, the cells were grown on SCD agar (FUJIFILM Wako Pure Chemical Co.) at 32.5°C.

### Determination of MIC and MBC

The MIC and MBC were determined using the broth dilution method (46) with minor modifications. Test solutions were prepared at the maximum concentration in SCD broth (FUJIFILM Wako Pure Chemical Co.) such that the final concentrations of PE (FUJIFILM Wako Pure Chemical Co.) and EDTA disodium (DOJINDO) were 2.0% and 5.0%, respectively, and the pH was adjusted to pH 7.0. Stepwise 1.25-fold dilutions were then prepared in 96-well microplates using SCD broth. Bacterial colonies were collected from SCD agar and suspended in SCD broth at an optical density at 600 nm (OD_600_) of 0.011. A 20 μL aliquot of the resulting suspension was mixed with 200 μL of the serially diluted test solution to obtain approximately 10^5^ CFU/mL per well. The MICs were defined as the lowest concentrations of PE or EDTA that inhibited visible growth, determined as an increase in turbidity by a factor of 100 (OD_600_ ≥ 0.1) after 24 h of incubation. To determine MBCs, 10 μL of the MIC test solution was mixed and subcultured in 90 or 990 μL of fresh SCD broth for PE or EDTA, respectively. After overnight culture, the lowest concentration showing no visible growth in the subcultures was considered the MBC. All experiments were performed in triplicate.

### Time–kill curve assay

The cell suspension and designated concentrations of PE and EDTA were prepared as described above. A 20 μL aliquot of the cell suspension was mixed with 200 μL of test solution in 96-well plates. Aliquots were withdrawn at specific time points, diluted with lecithin and polysorbate 80 (FUJIFILM Wako Pure Chemical Co.), and spread onto SCD agar plates. CFUs were counted after incubating the plates for 30–48 h. All experiments were performed in triplicate.

### Fabrication of the microdevice

The microdevice consisted of a polydimethylsiloxane (PDMS) chip, patterned coverslips with cell-trapping microchambers, and a semi-permeable cellulose membrane. Each component was prepared as previously described (27, 28). The PDMS chip and patterned coverslips were fabricated using standard photolithographic technologies by Kyodo International, Inc. (Kawasaki, Japan). To create the first component, the PDMS chip, a silicon wafer was spin-coated with negative photoresist SU-8 3005 to a thickness of 300 μm and exposed to UV light through a serpentine design mask. The wafer was then used as a mold to cast PDMS (1:10 elastomer to curing agent). After curing, holes were punched in the 5 mm thick PDMS chip using a 2 mm diameter punch. Silicon tubes (0.76 mm inner diameter) were connected to the inlet and outlet and sealed using PDMS as an adhesive. To create the second component, the patterned coverslips, SU-8 was used to coat 25 mm diameter coverslips to a thickness of approximately 700 nm, which were then patterned by photolithography using a mask containing cell-trapping microchambers. To create the third component, the membrane, semi-permeable cellulose membrane with a molecular weight cutoff of ≤25 kDa (pre-treated Spectra/Por 7 dialysis tubing; Spectrum Laboratories, Inc., CA, USA) was cut into 25 mm diameter circular sections using a Thomson blade. Finally, all three components were assembled between a transparent acrylic cover and a base adapter with screws.

### Time-lapse imaging of single cells

The cells grown on agar plates were suspended in saline to an OD_600_ of 0.2. A saline-moistened cellulose semi-permeable membrane was placed over the flow path of a sterilized PDMS chip, and 10 µL of the cell suspension was spread onto it. The microdevices were then assembled such that the surface of the cell suspension was aligned with that of the patterned coverslip placed on the base adapter. The inlet tube was connected to a 50 mL syringe, and the outlet tube was connected to a waste tank. SCD broth containing PE, with or without EDTA, was pumped through the PDMS chip at a flow rate of 25 μL/min. To monitor cell death, 1 μg/mL PI (Molecular Probes) was added to the medium. Bright-field and fluorescence images were recorded at 100× magnification, with excitation at 488 nm and emission at 565–700 nm, using an LSM 880 confocal laser scanning microscope (Carl Zeiss). Images were captured at approximately 50 XY points at 5-min intervals over a ~20 h period. Each experimental condition was repeated at least twice.

### Image analysis

Image analysis was performed using ImageJ software (National Institute of Health). The single-cell lifespan was defined as the time interval between the frame in which the cell was born, identified by a popping event (33), and the frame in which the cell died, detected by PI staining or cell lysis. The single-cell division rate was defined as the reciprocal of the time interval between the successive popping events. All cell counts and the classification of events (birth, division, and death) were performed manually by visual inspection of the time-lapse image series using ImageJ.

### Statistical analysis

Statistical tests were performed using GraphPad Prism version 7.05 (GraphPad Software Inc.). Two-way repeated-measures analysis of variance was used to compare kill curves. The Kolmogorov–Smirnov test was applied to compare the number of cell divisions. A value of *P* < 0.05 was considered statistically significant.

## Data Availability

The whole-genome sequence of *S. ureilyticus* KMC153 has been deposited in the DDBJ Sequence Read Archive under BioProject accession number PRJDB38213. The microscopy images have been deposited in Figshare (DOI: 10.6084/m9.figshare.31165393).

## SUPPLEMENTAL MATERIAL

Supplemental material spectrum.03807-25-s0001.docx Legends for all supplemental figures and movies.

Fig. S1 spectrum.03807-25-s0002.tif Dose-dependent suppression of cell division.

Fig. S2 spectrum.03807-25-s0003.tif Generation-wise decline in the proportion of dividing progeny under PE exposure.

Fig. S3 spectrum.03807-25-s0004.tif Distribution of single-cell division rate in SCD medium.

Fig. S4 spectrum.03807-25-s0005.tif Pedigree trees of 29 mother cells showing varying numbers of successive divisions under 1.2% PE.

Movie S1 spectrum.03807-25-s0006.avi Representative time-lapse movie of 1.2% PE treatment.

Movie S2 spectrum.03807-25-s0007.avi Representative time-lapse movie of the combined 1.0% PE and 0.14% EDTA treatment.

ASM does not own the copyrights to Supplemental Material that may be linked to, or accessed through, an article. The authors have granted ASM a non-exclusive, world-wide license to publish the Supplemental Material files. Please contact the corresponding author directly for reuse.
