# Supplementary material for: EDTA suppresses bacterial perseverance to 2-phenoxyethanol
Source: Microbiol Spectr. 2026 Apr 30;14(6):e03807-25. doi: 10.1128/spectrum.03807-25 (PMC13228015; doi:10.1128/spectrum.03807-25)
Supplement: Supplemental material — Legends for all supplemental figures and movies. [file spectrum.03807-25-s0001.docx]

**EDTA suppresses bacterial perseverance to 2-phenoxyethanol**

**SUPPLEMENTAL LEGENDS**

**MOV S1**

Representative time-lapse movie of 1.2% PE treatment. Cells were cultured at 32.5 °C in a custom-made microdevice with a continuous flow of SCD medium containing 1.2% PE. Phase-contrast and fluorescence images were captured at 5-min intervals using an oil immersion objective (100× magnification). PI (1 µg/mL) was added to detect cell death.

**MOV S2**

Representative time-lapse movie of the combined 1.0% PE and 0.14% EDTA treatment. Cells were cultivated at 32.5 °C in a custom-made microdevice with a continuous flow of SCD medium containing 1.0% PE and 0.14% EDTA. Phase-contrast and fluorescence images were captured at 5-min intervals using an oil immersion objective (100× magnification). PI (1 µg/mL) was added to detect cell death.

**FIG S1** Dose-dependent suppression of cell division. Cells were exposed to various concentrations of PE (1.2%, orange; 1.3%, red; 1.6%, dark red), 1.0% PE + 0.14% EDTA (gray), or SCD medium (control, black open symbol). Normalized cell numbers over time are shown. Cell numbers represent the sum of dead and intact cells, plotted as points with linear connecting lines. The number of mother cells at the initial time points was 32 (SCD medium), 239 (1.2% PE), 88 (1.3% PE), 120 (1.6% PE), and 228 (1.0% PE + 0.14% EDTA).

**FIG S2** Generation-wise decline in the proportion of dividing progeny under PE exposure. Cells were exposed to 1.2% PE, and the proportion of dividing cells in each generation was calculated. The numbers of cells analyzed for generations G1–G5 were 99, 149, 181, 146, and 65, respectively.

**FIG S3** Distribution of single-cell division rate in SCD medium. Division rate (h⁻¹) is defined as the reciprocal of the interdivision time. Each point represents an individual cell, and the average division rate was 1.38 ± 0.38 h⁻¹, with a minimum of 0.47 h⁻¹ and a maximum of 2.40 h⁻¹.

**FIG S4** Pedigree trees of 29 mother cells showing varying numbers of successive divisions under 1.2% PE. Branches indicate cell divisions, and boundaries that change from black to red indicate cell death.
