# Supplementary figures and images for: EDTA suppresses bacterial perseverance to 2-phenoxyethanol
Source: Microbiol Spectr. 2026 Apr 30;14(6):e03807-25. doi: 10.1128/spectrum.03807-25 (PMC13228015; doi:10.1128/spectrum.03807-25)

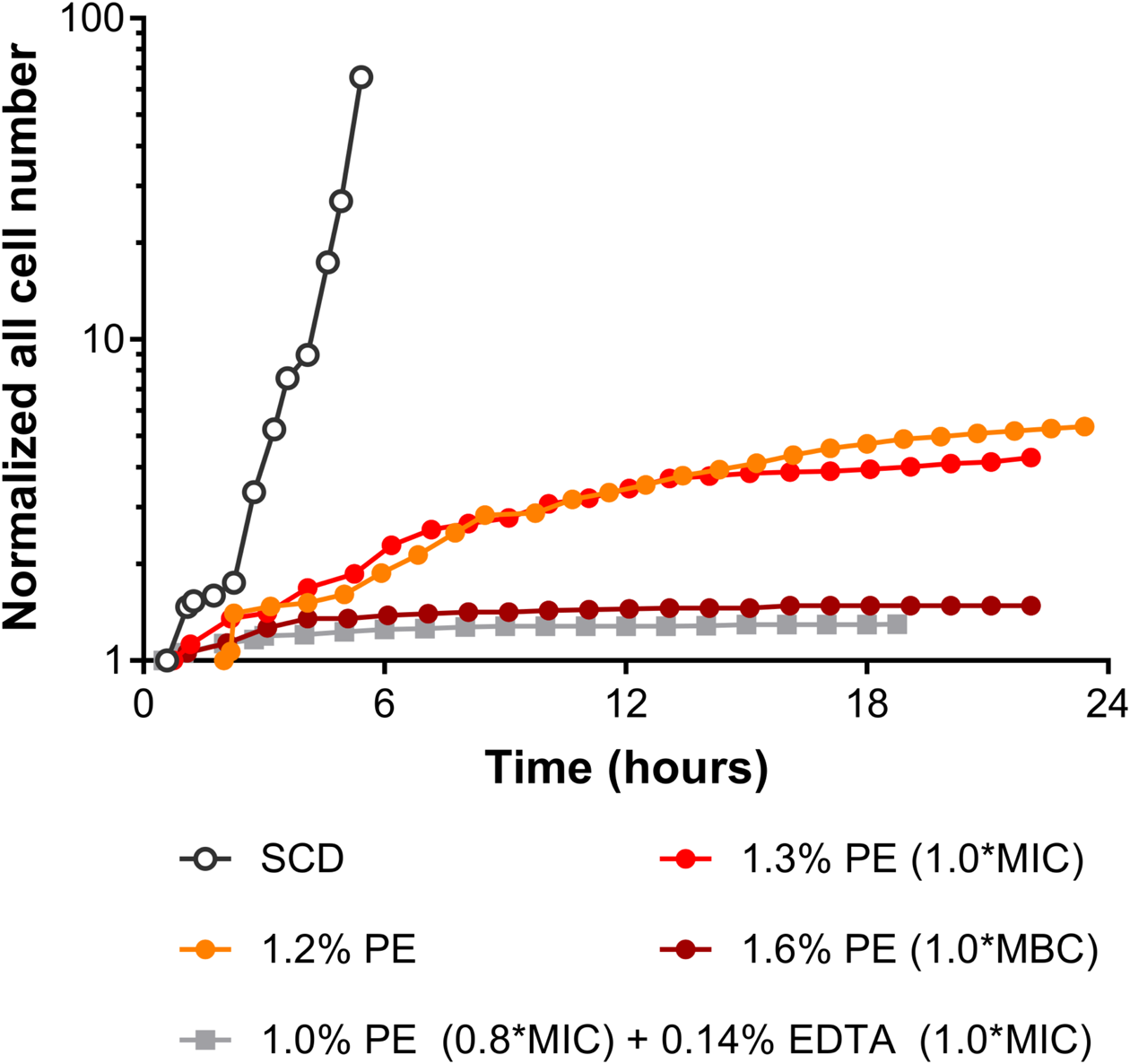

Supplement: Fig. S1 — Dose-dependent suppression of cell division. [file spectrum.03807-25-s0002.tif]

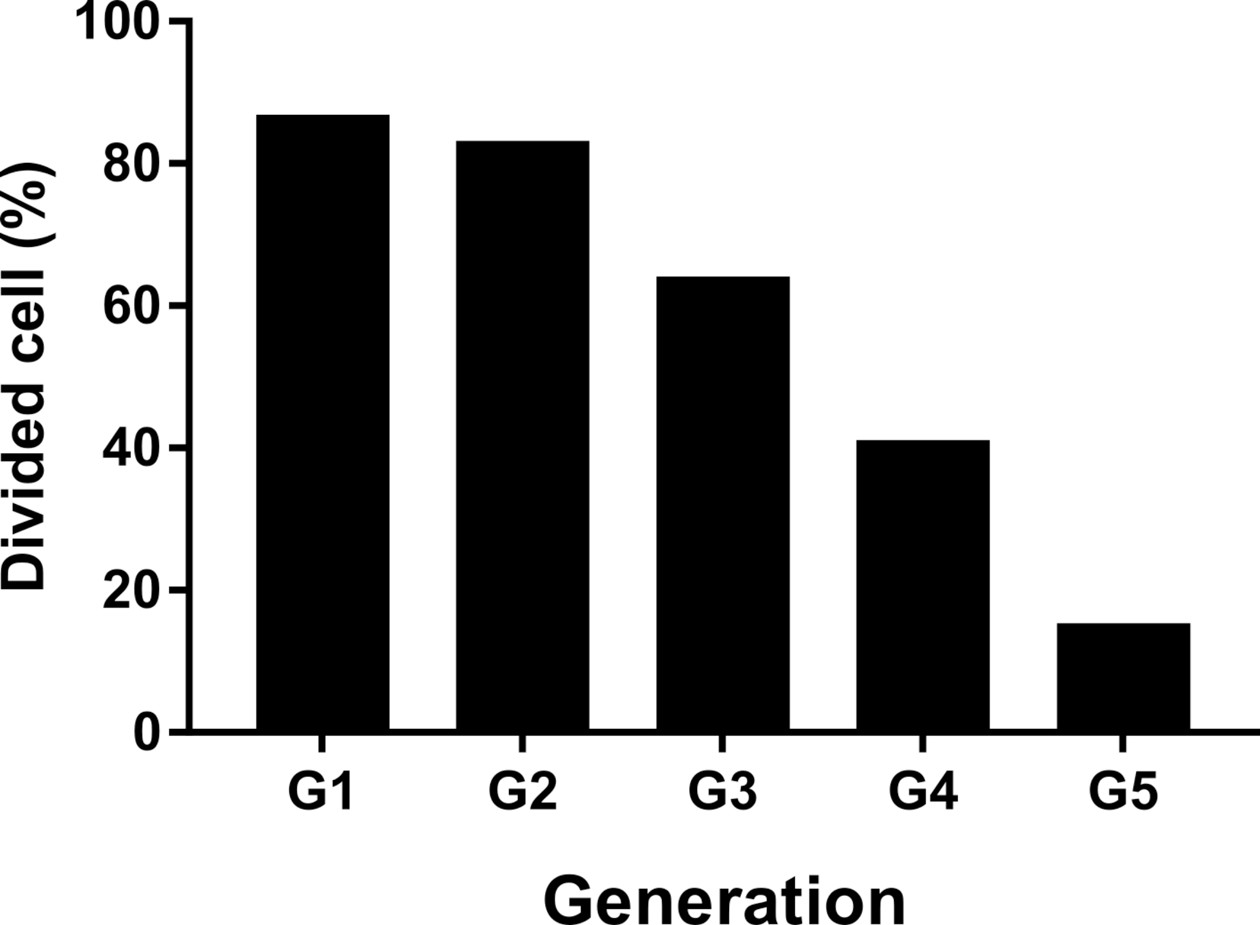

Supplement: Fig. S2 — Generation-wise decline in the proportion of dividing progeny under PE exposure. [file spectrum.03807-25-s0003.tif]

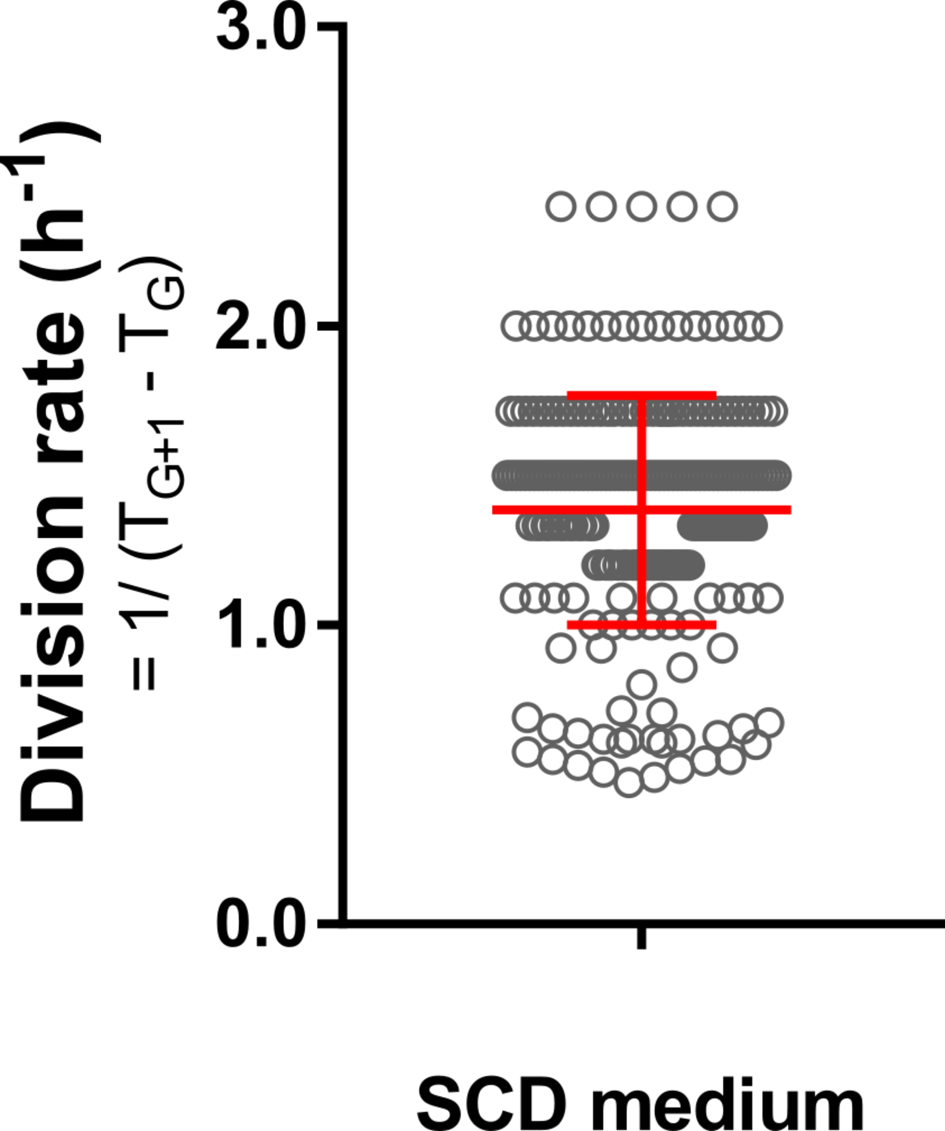

Supplement: Fig. S3 — Distribution of single-cell division rate in SCD medium. [file spectrum.03807-25-s0004.tif]

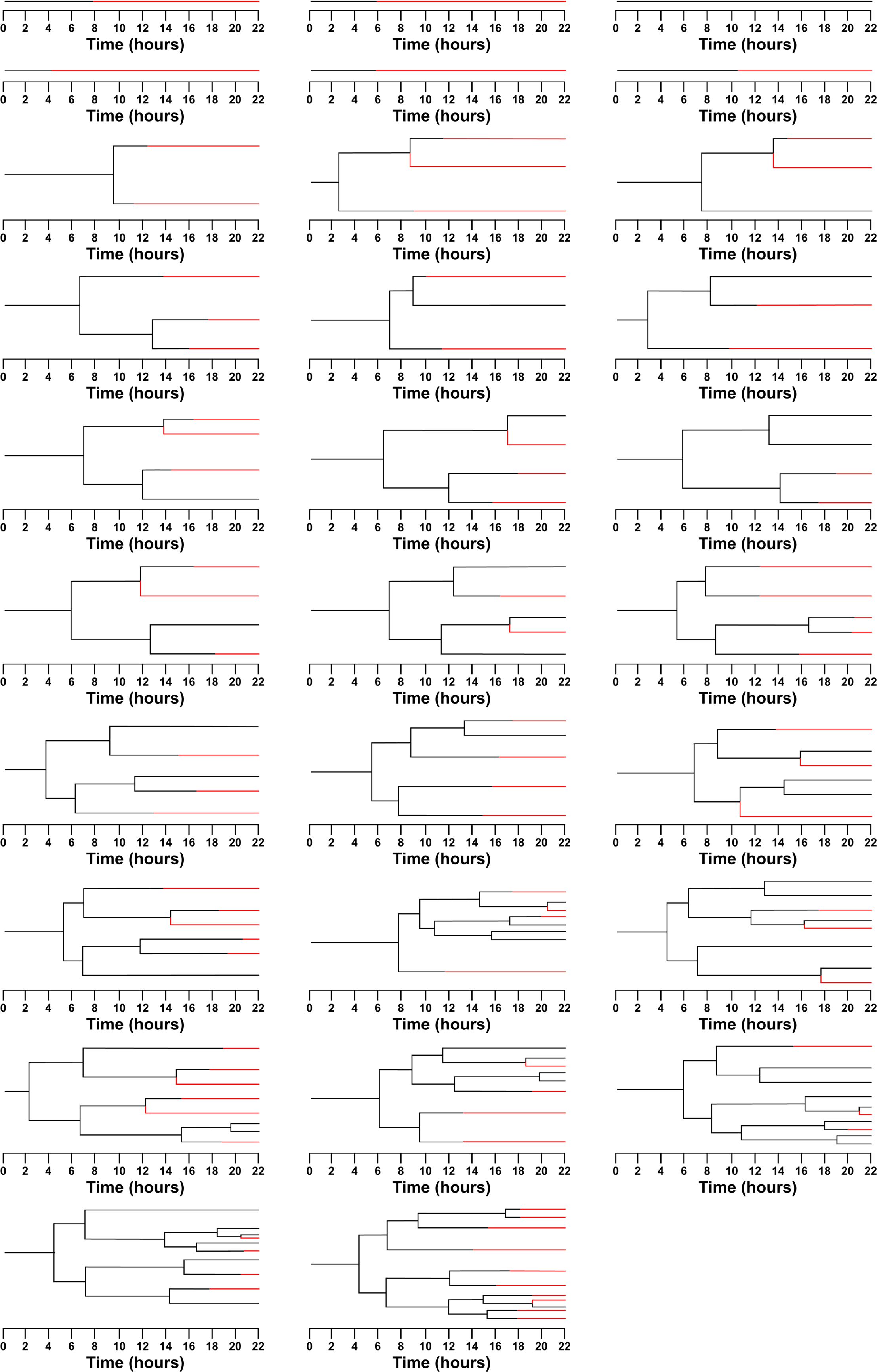

Supplement: Fig. S4 — Pedigree trees of 29 mother cells showing varying numbers of successive divisions under 1.2% PE. [file spectrum.03807-25-s0005.tif]
